# Three-dimensional spatial modeling of spines along dendritic networks in human cortical pyramidal neurons

**DOI:** 10.1371/journal.pone.0180400

**Published:** 2017-06-29

**Authors:** Laura Anton-Sanchez, Pedro Larrañaga, Ruth Benavides-Piccione, Isabel Fernaud-Espinosa, Javier DeFelipe, Concha Bielza

**Affiliations:** 1 Departamento de Inteligencia Artificial, Escuela Técnica Superior de Ingenieros Informáticos, Universidad Politécnica de Madrid, Madrid, Spain; 2 Laboratorio Cajal de Circuitos Corticales, Centro de Tecnología Biomédica, Universidad Politécnica de Madrid, Madrid, Spain; 3 Instituto Cajal, Consejo Superior de Investigaciones Científicas, Madrid, Spain; Universidad de Salamanca, SPAIN

## Abstract

We modeled spine distribution along the dendritic networks of pyramidal neurons in both basal and apical dendrites. To do this, we applied network spatial analysis because spines can only lie on the dendritic shaft. We expanded the existing 2D computational techniques for spatial analysis along networks to perform a 3D network spatial analysis. We analyzed five detailed reconstructions of adult human pyramidal neurons of the temporal cortex with a total of more than 32,000 spines. We confirmed that there is a spatial variation in spine density that is dependent on the distance to the cell body in all dendrites. Considering the dendritic arborizations of each pyramidal cell as a group of instances of the same observation (the neuron), we used replicated point patterns together with network spatial analysis for the first time to search for significant differences in the spine distribution of basal dendrites between different cells and between all the basal and apical dendrites. To do this, we used a recent variant of Ripley’s *K* function defined to work along networks. The results showed that there were no significant differences in spine distribution along basal arbors of the same neuron and along basal arbors of different pyramidal neurons. This suggests that dendritic spine distribution in basal dendritic arbors adheres to common rules. However, we did find significant differences in spine distribution along basal versus apical networks. Therefore, not only do apical and basal dendritic arborizations have distinct morphologies but they also obey different rules of spine distribution. Specifically, the results suggested that spines are more clustered along apical than in basal dendrites. Collectively, the results further highlighted that synaptic input information processing is different between these two dendritic domains.

## Introduction

Many types of real-world events are constrained by networks, such as stores located alongside streets, traffic accidents on roads, street crime sites, etc. These events are called *network events*. Network spatial analysis refers to statistical and computational methods for analyzing events occurring on or along networks. Most of these methods have been developed by Okabe and collaborators [1] and include techniques similar to the methods used in traditional spatial analysis but taking into account the network topology. The main difference from traditional spatial analysis using Euclidean distances is that network spatial analysis measures shortest path distances. Shortest path distances are much harder to calculate because they require network topology management. If traditional spatial analysis assuming a plane with Euclidean distances [2] is applied to network events, then we are likely to draw false conclusions due to short-range clustering (due to the concentration of events, for example, on a road) and/or long-range regularity (for example, due to the separation of different roads).

Existing techniques for network spatial analysis assume that the network is two dimensional. In this paper, we extend these techniques to the 3D space in order to model the spatial distribution of dendritic spines (for simplicity, spines) of pyramidal neurons, the principal building blocks of the cerebral cortex. Since spines are the main postsynaptic target of excitatory synapses in the cerebral cortex, many researchers are interested in ascertaining their spatial distribution within the two main dendritic arbors of pyramidal cells: the apical and basal dendritic trees. As previously described [3], the apical arbor stems from a main apical shaft whose origin is the upper pole of the pyramidal cell body. This apical dendrite is radially directed towards the pia mater and gives off a number of oblique branches. A system of large basal dendrites (generally, from three to six) emerges from the base of the pyramidal cell body and is directed laterally or downward. Generally speaking, proximal dendrites receive excitatory inputs from local sources (collaterals in the same area or from an adjacent area), whereas the distal apical tuft receives inputs from more distant cortical and thalamic locations; while the proximal portions of pyramidal cell dendrites are devoid of spines (approximately 10-15 *μ*m from the cell body), there is a progressive increase in the density of spines. The highest densities are found at variable distances from the soma, depending on the cortical area and species. In the human temporal cortex, the highest density is found at a distance of 75-125 *μ*m from the cell body. Thereafter, there is a progressive decrease towards the distal tips of dendrites, where the density is again low. Spines must necessarily lie on the dendritic shaft. Therefore, the application of network spatial analysis is appropriate. Some recent research [4, 5] also used network spatial analysis to analyze spine distribution along dendrites. However, using the justification that neurons in cell culture *in vitro* are almost flat, they ignored the third dimension and used a 2D projection. To the best of our knowledge, this is the first time that 3D network spatial analysis has been applied.

Taking advantage of the fact that we had several dendritic arborizations from each pyramidal cell, which can be treated as a group of instances of the same neuron, we also used replicated point patterns to detect differences and similarities between different pyramidal neurons and between apical and basal dendrites. The number of works related to applications to neuroanatomical data using replicate spatial patterns techniques is growing strongly [6–13]. These techniques are used here together with network spatial analysis for the first time.

## Materials and methods

### Cell reconstruction

**Fig 1:**
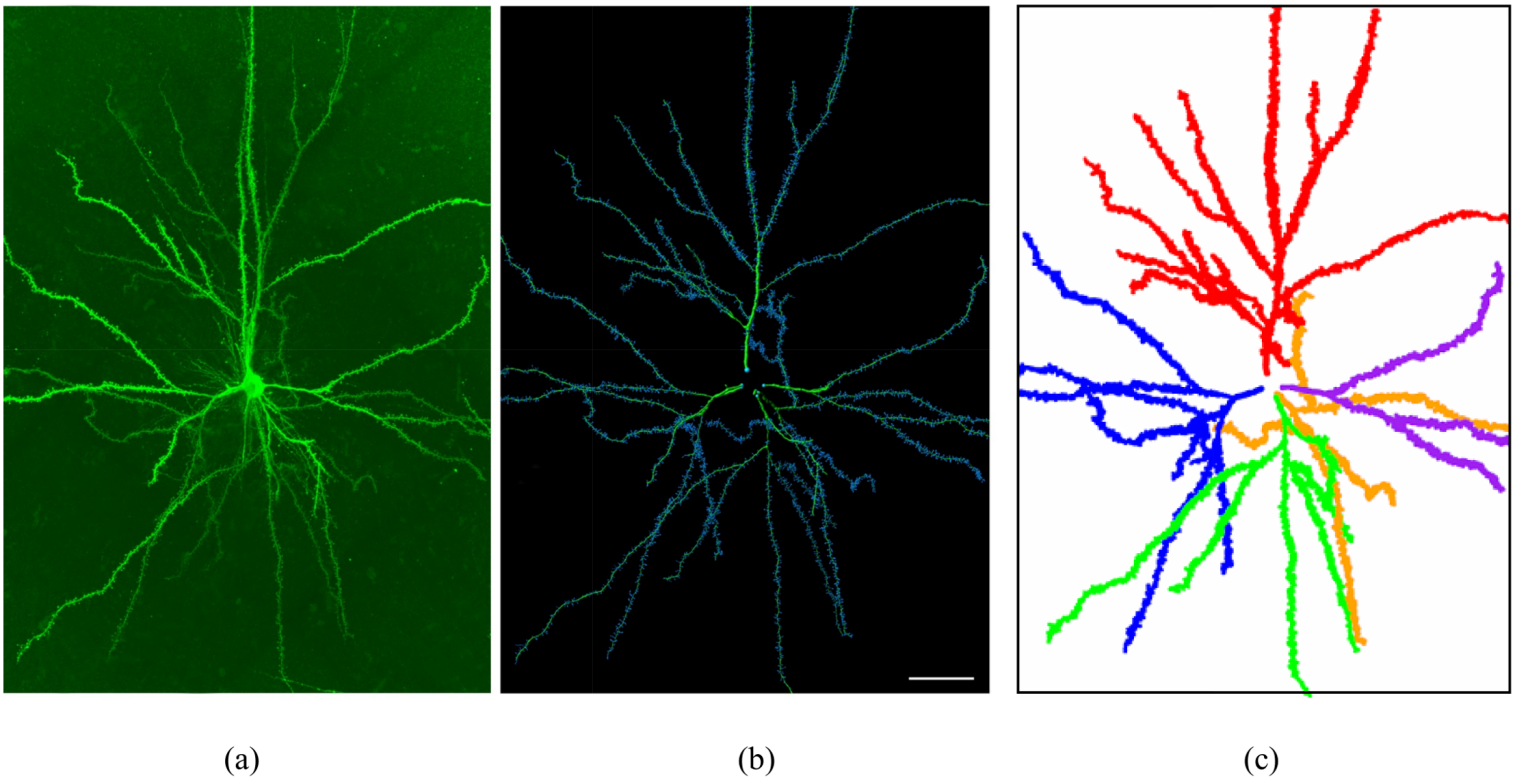
Example of one of the analyzed pyramidal neurons. (a) Confocal microscopy image of an intracellularly injected layer III pyramidal neuron of the human temporal cortex (Neuron 1 in Tables 1 and 2), visualized in 3D from high-resolution confocal stacks of images. (b) 3D reconstruction of the complete morphology of the cell shown in (a). (c) 3D reconstruction of the same neuron showing the apical dendrite in red and the four reconstructed basal dendrites in blue, green, orange and purple. We use the blue basal tree in (c) throughout the manuscript to illustrate the analysis performed. Scale bar (in (b)): 50 *μ*m.

**Fig 2:**
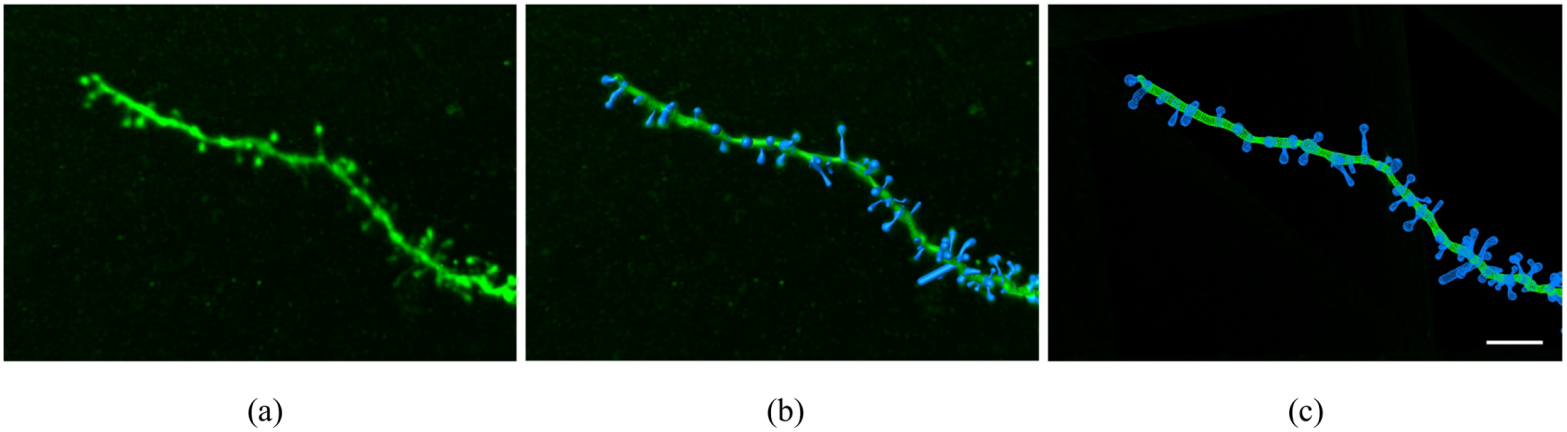
Example of basal dendritic segment. (a) High magnification confocal microscopy image showing a basal dendritic segment from Neuron 1. (b, c) Reconstruction of the dendritic shaft and spines shown in (a) in a solid (b) and mesh (c) view.

In this paper, we analyzed five detailed and complete reconstructions of adult human pyramidal neurons that were intracellularly injected with Lucifer Yellow (LY) in layer III of the temporal cortex (area 20 of Brodmann) from two human males (aged 40 and 66) obtained at autopsy (2–3 h post-mortem) that died in traffic accidents. The brain samples were obtained following the guidelines and with the approval of the Institutional Ethical Committee. The tissue from these human brains has been used and described as histologically normal in previous studies [14, 15]. Detailed information regarding tissue preparation, injection methodology and immunohistochemistry processing was described in [16, 17]. The injected cells were fully imaged at high magnification using the tile scan mode in a Leica TCS 4D confocal scanning laser attached to a Leitz DMIRB fluorescence microscope (Fig 1). Consecutive stacks of images at high magnification (x63 glycerol) were acquired to capture dendrites along the apical and basal dendritic arbors. The dendritic arborization was reconstructed using Imaris 7.6.5, Filament Tracer module software (Bitplane AG, Zurich, Switzerland). Therefore, we were able to place their spines, adjusting the position, length and volume of each spine individually (Fig 2). We analyzed a total of more than 32,000 spines, 44% in apical dendrites and 56% in basal dendrites. In all cases, brain tissue donation, processing and use for research are performed in compliance with published protocols [18], which include the obtaining of informed consent for brain tissue donation from living donors, and the approval of the whole donation process by the Spanish Research Council CSIC Ethics Committee and the Ethics Committee of the “Banco de Tejidos Fundación CIEN” (BTFC; Centro Alzheimer, Fundación Reina Sofía, Madrid, Spain), following national laws and international ethical and technical guidelines on the use of human samples for biomedical research purposes.

### Spatial analysis along networks

A linear network *L* in $R^{3}$ is defined as the union of a finite collection of line segments *l*_*i*_ in $R^{3}$ (*i* = 1, …, *m*), where a line segment with endpoints $u\in R^{3}$ and $v\in R^{3}$ is defined as [*u*, *v*] = {*su* + (1 − *s*)*v*: 0 ≤ *s* ≤ 1}. The shortest path distance between two points *u* and *v* located in *L*, *d*_*L*_(*u*, *v*), is the minimum length of all paths along the network from *u* to *v*. If there are no paths from *u* to *v* (the network is not connected), then *d*_*L*_(*u*, *v*) = ∞. A network that has no cycles is called acyclic network or tree. Each dendritic arborization is a tree in which all points are connected.

Let ***X*** be a point process on a linear network *L*. A realization of ***X*** is a finite set ***x*** = {*x*_1_, …, *x*_*n*_} of distinct points *x*_*i*_ located in *L*, where *n* ≥ 0 is not fixed in advance. Each point *x*_*i*_ is called *network event*. The intensity function λ(*u*), *u* ∈ *L* of a point process ***X*** on a linear network *L*, is the expected number of points per unit length in the network in the vicinity of *u*. The intensity of the homogeneous Poisson process or Complete Spatial Randomness (CSR) is constant λ(*u*)≡λ, where $\hat{\lambda }=n/\left|L\right|$ is an unbiased estimator of the intensity, *n* being the number of points in ***x*** and |*L*| being the total length of all line segments in *L*. The general intensity function of a point process ***X*** on a linear network can be estimated using kernel smoothing estimators [19].

One of the most commonly used summary characteristics in spatial point pattern analysis is Ripley’s *K* function [20]. As described in detail previously [20], for a stationary process (i.e., statistically invariant under translations), Ripley’s *K* function for a distance *d*, *K*(*d*), is the expected number of other points of the process within a distance *d* of a typical point *u* of the process divided by the intensity. Patterns where the distances between points are shorter (larger) than expected in a random pattern of the same intensity are known as clustered (regular) patterns, and the curve of their *K* function will be shifted to the left (right) with respect to that of a CSR pattern of the same intensity.

Let *L* be a linear network with events at locations *x*_1_, …, *x*_*n*_. A network *K* function analogous to Ripley’s *K* function is developed in [21], where the shortest path distances in the network *d*_*L*_(*x*_*i*_, *x*_*j*_) replace the Euclidean distances. This function is estimated as:

$${\hat{K}}_{net}\left(d\right)=\frac{\left|L\right|}{n(n-1)}\sum_{i=1}^{n}\sum_{j\neq i}1\left\{d_{L}\left(x_{i},x_{j}\right)\leq d\right\}\text{,}$$ (1)

where 1{⋅} denotes the indicator function. As shown in [22], the estimated value of the network *K* function depends on the geometry of the network. Therefore, the network *K* functions of different networks are not directly comparable.

The solution proposed in [22] was a geometrically corrected version of the network *K* function, *K*_*L*_, that compensated for the geometry of the network. The empirical estimator of *K*_*L*_ is intrinsically corrected for edge effects, and its variance is approximately stabilized. The geometrically corrected empirical *K* function for a distance *d* is defined as:

$${\hat{K}}_{L}\left(d\right)=\frac{\left|L\right|}{n(n-1)}\sum_{i=1}^{n}\sum_{j\neq i}\frac{1\{d_{L}\left(x_{i},x_{j}\right)\leq d\}}{m(x_{i},d_{L}\left(x_{i},x_{j}\right))}$$ (2)

for 0 ≤ *d* ≤ *R*, where *m*(*u*, *t*) = #{*v* ∈ *L*: *d*_*L*_(*u*, *v*) = *t*} is the number of points located in *L* lying at the exact distance *t* from the point *u* measured by the shortest path, and *R* = sup{*t*: *m*(*u*, *t*) > 0 for all *u* ∈ *L*} is the *circumradius* of the network, i.e., the radius of the smallest disc that contains the entire network, as explained in [22].

For a homogeneous Poisson process on *L*, *K*_*L*_(*d*) = *d* for all 0 ≤ *d* ≤ *R*. This provides a simple benchmark for completely spatial random point patterns on a linear network and also allows comparison between geometrically corrected *K* functions obtained from different point patterns in different networks.

The inhomogeneous version of Ripley’s *K* function is introduced in [23] for non-constant intensity spatial point processes. The contribution to the inhomogeneous *K* function of each pair of points *x*_*i*_ and *x*_*j*_ is weighted by 1/(λ(*x*_*i*_)λ(*x*_*j*_)). Consequently, the properties of the inhomogeneous *K* function are very similar to the original version of Ripley’s *K* function. The inhomogeneous network *K* function is similarly defined in [22] for a spatial point process on a linear network, estimated as:

$${\hat{K}}_{LI}\left(d\right)=\frac{1}{\sum_{i}1/\hat{\lambda }\left(x_{i}\right)}\sum_{i=1}^{n}\sum_{j\neq i}\frac{1\{d_{L}\left(x_{i},x_{j}\right)\leq d\}}{\hat{\lambda }\left(x_{i}\right)\hat{\lambda }\left(x_{j}\right)m\left(x_{i},d_{L}\left(x_{i},x_{j}\right)\right)}\text{,}$$ (3)

where $\hat{\lambda }\left(\cdot \right)$ is the estimated intensity function.

To test whether the deviation between two summary functions, usually between the empirical summary function and the summary function of the model to be tested, is statistically significant, the standard approach is to use a Monte Carlo test based on envelopes of the summary function obtained from simulated point patterns. We used global envelopes since the range of spatial interaction was unknown. We calculated the envelopes by generating 19 simulations of the model to be tested, computing the summary functions of the simulated patterns. The global envelope has constant width 2*w*_*max*_, where *w*_*max*_ is the maximum absolute difference between the theoretical value of the summary function of the model to be tested and any of the summary functions of the simulated patterns. This corresponds to a Monte Carlo test with significance level 1/(1+19) = 0.05 [24]. If the empirical summary function is completely contained in the envelope, the model is not rejected.

**Fig 3:**
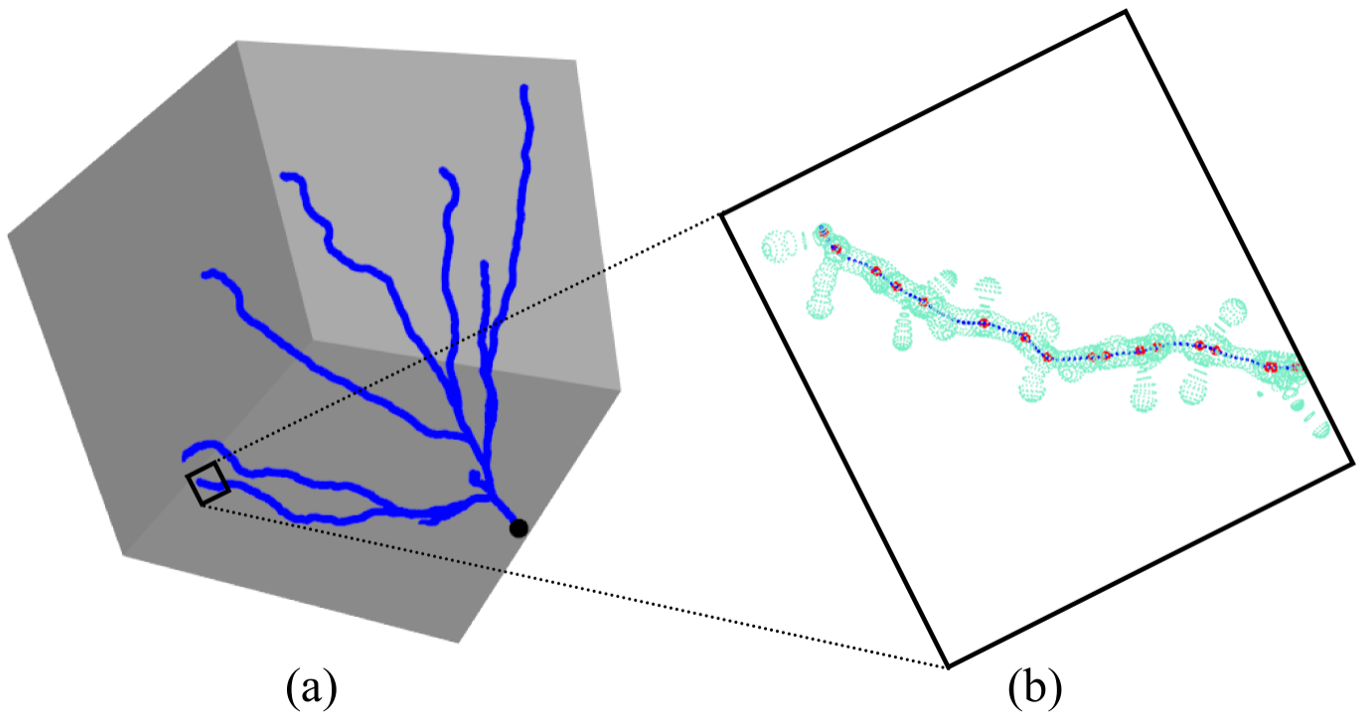
First basal arborization of Neuron 1 illustrating the analysis (some of its characteristics are shown in Table 2). (a) 3D representation of the basal network. The tree root is shown in black. (b) Zoom of a small part of the same dendrite (end of the dendritic segment shown in Fig 2) to illustrate the computation of the dendrite axis (i.e., the network in dark blue) and the attachment points of the spines (network events in red) from the reconstruction provided in the *.vrml* file (light blue).

Existing computational techniques for spatial analysis along networks assume that the network is 2D [1, 25]. Although dendritic networks are 3D, recent research analyzing the distribution of spines along dendritic arborizations [4, 5] ignored the third dimension, arguing that neurons in cell culture *in vitro* are more or less flat. They used a 2D projection to represent the spatial layout of the dendrites. In our case, cell reconstructions have a third dimension that should not be overlooked. For example, Fig 3a shows the first basal dendrite of the first analyzed pyramidal neuron, clearly illustrating that the dendritic tree is not flat. We extended the functionality provided by the **spatstat** package [26, 27] designed to manage 2D networks in order to handle 3D networks. Thus, we have performed the first spatial analysis along 3D networks. Eqs (1)–(3) are applicable to 3D linear networks, although key values like *d*_*L*_(*x*_*i*_, *x*_*j*_) or *m*(*u*, *t*) are much harder to compute taking into account the third dimension.

From the specifications of pyramidal neurons in *.vrml* format, we obtained the 3D axes of the dendritic arborizations and the spine attachment points (network and network events in the model, respectively, see Fig 3b). After processing the *.vrml* files using R software, we used the **spatstat** package and the extensions that we implemented for the 3D analysis in order to represent the networks and analyze the distribution of the spines along dendritic networks.

### Replicated spatial point patterns

Replicated point patterns are a collection of point patterns that can be regarded as the realizations of the same point process [6]. For replication in groups with which we are concerned, there are *g* different experimental groups. In group *i* (*i* = 1, …, *g*), we observe *m*_*i*_ point patterns that can be regarded as independent replicates within this group. Replication provides for the analysis of the differences in spatial point patterns between and within groups for decision making on whether there are statistically significant differences between groups. We had several basal dendritic trees from each pyramidal neuron that can be regarded as replicates of the same observation (the neuron). By conducting an analysis in the context of replicated point patterns, we investigated whether there were significant differences between the basal arborizations of the same pyramidal neuron and between different pyramidal neurons, that is, we performed a study with *g* = 5 groups, where each group was composed of the basal dendrites of each pyramidal neuron. We were also interested in analyzing whether there were significant differences in the distribution of spines along the apical and basal networks, that is, in performing a study with *g* = 2 groups (one group with apical dendrites and the other with all basal dendrites of all neurons).

We tested the null hypothesis of similarity between groups with the studentized permutation test proposed in [28]. Suppose we have *g* groups of point patterns, with *m*_1_, …, *m*_*g*_ point patterns each. The patterns in group *i* (*i* = 1, …, *g*) are named ***x***_*i*1_, …, ***x***_*im*_*i*__. The test proposed in [28] compares the means of groups corresponding to estimates ${\hat{T}}_{ij}\left(d\right)$, where *T* is the summary function of pattern ***x***_*ij*_ in an interval [*d*_0_, *d*_1_] for *d*, with the test statistic

$$H=\sum_{1\leq i\leq j\leq g}\int_{d_{0}}^{d_{1}}\frac{{\left({\bar{T}}_{i}\left(d\right)-{\bar{T}}_{j}\left(d\right)\right)}^{2}}{\frac{1}{m_{i}}\bar{s_{i}^{2}}+\frac{1}{m_{j}}\bar{s_{j}^{2}}}\text{d}d\text{,}$$ (4)

where ${\bar{T}}_{i}\left(d\right)=\left(1/m_{i}\right)\sum_{j=1}^{m_{i}}{\hat{T}}_{ij}\left(d\right)$ is the mean in group *i* and

$$\bar{s_{i}^{2}}=\frac{1}{d_{1}-d_{0}}\int_{d_{0}}^{d_{1}}\frac{1}{m_{i}-1}\sum_{j=1}^{m_{i}}{\left({\hat{T}}_{ij}\left(d\right)-{\bar{T}}_{i}\left(d\right)\right)}^{2}\text{d}d$$

are the estimated within-group variances of the estimates. The test is performed by calculating *H* statistic for the observed data and for a large number of random permutations of the set of point patterns, and then computing the *p*-value ranking the observed value of the test statistic among the corresponding permutation values of the test statistic.

As mentioned, the geometrically corrected *K* function compensates for the geometry of the network, whereby the corrected *K* functions obtained from different point patterns in different networks are directly comparable. Therefore, this is the first time that the geometrically corrected *K* function (Eqs (2) or (3)) has been applied in the context of replicated point patterns to compare different groups of 3D point patterns on linear networks. We used the studentized permutation test provided in **spatstat**, which we expanded to be used with the *K* function on linear networks. We used 1000 permutations for the test (default value).

## Results

Table 1 shows some important characteristics of the apical dendrites of each of the five analyzed pyramidal neurons: number of spines, total length of the network, average number of points per unit length in the network, *circumradius* and number of branching points (complexity measure of the dendritic tree). Table 2 shows the same information for basal dendrites, grouped according to the pyramidal neuron to which they belong. Apical dendrites are clearly more complex than basal dendrites, as a comparison of the mean number of characteristics shown in Tables 1 and 2 patently shows. While the mean number of spines in apical dendrites is 2845, it is 1074 in basal dendrites. The mean length is also much greater in apical than in basal arborizations: 2497.25 *μ*m and 951.85 *μ*m, respectively. The same applies to the mean number of branching points (20 in apical networks vs 6 in basal networks).

**Table 1:** Description of the analyzed apical dendrites. The table shows the number of spines *n*, total length of the network |*L*| (in *μ*m) average number of points per unit length in the network *n*/|*L*|, *circumradius* *R* (in *μ*m), and number of branching points in the dendrite #BP.

Neuron	*n*	|*L*|	*n*/|*L*|	*R*	# BP
1	2750	2182.42	1.26	237.48	16
2	3019	3073.93	0.98	325.49	22
3	2195	1852.01	1.19	231.55	18
4	2599	2123.39	1.22	261.55	19
5	3660	3254.50	1.12	332.11	23
Mean	2845	2497.25	1.16	277.64	20

**Table 2:** Description of the analyzed basal dendrites grouped by neuron. The table shows the number of spines *n*, total length of the network |*L*| (in *μ*m), average number of points per unit length in the network *n*/|*L*|, *circumradius* *R* (in *μ*m), and number of branching points in the dendrite #BP.

Neuron	*n*	|*L*|	*n*/|*L*|	*R*	# BP
1	1889	1527.45	1.24	257.74	8
1	584	615.79	0.95	208.72	3
1	1214	957.66	1.27	225.92	5
1	1272	1156.29	1.10	235.34	7
2	287	391.74	0.73	137.46	4
2	791	928.87	0.85	220.23	11
2	270	327.29	0.82	139.79	2
3	852	664.26	1.28	237.90	3
3	2149	1467.43	1.46	209.18	8
3	662	594.30	1.11	177.59	4
4	778	556.49	1.40	169.34	4
4	1487	1282.15	1.16	213.84	8
4	1004	834.83	1.20	202.17	5
5	2244	2231.19	1.01	309.20	14
5	978	879.04	1.11	204.76	7
5	1088	851.73	1.28	211.03	6
Mean	1074	951.85	1.10	208.49	6

**Fig 4:**
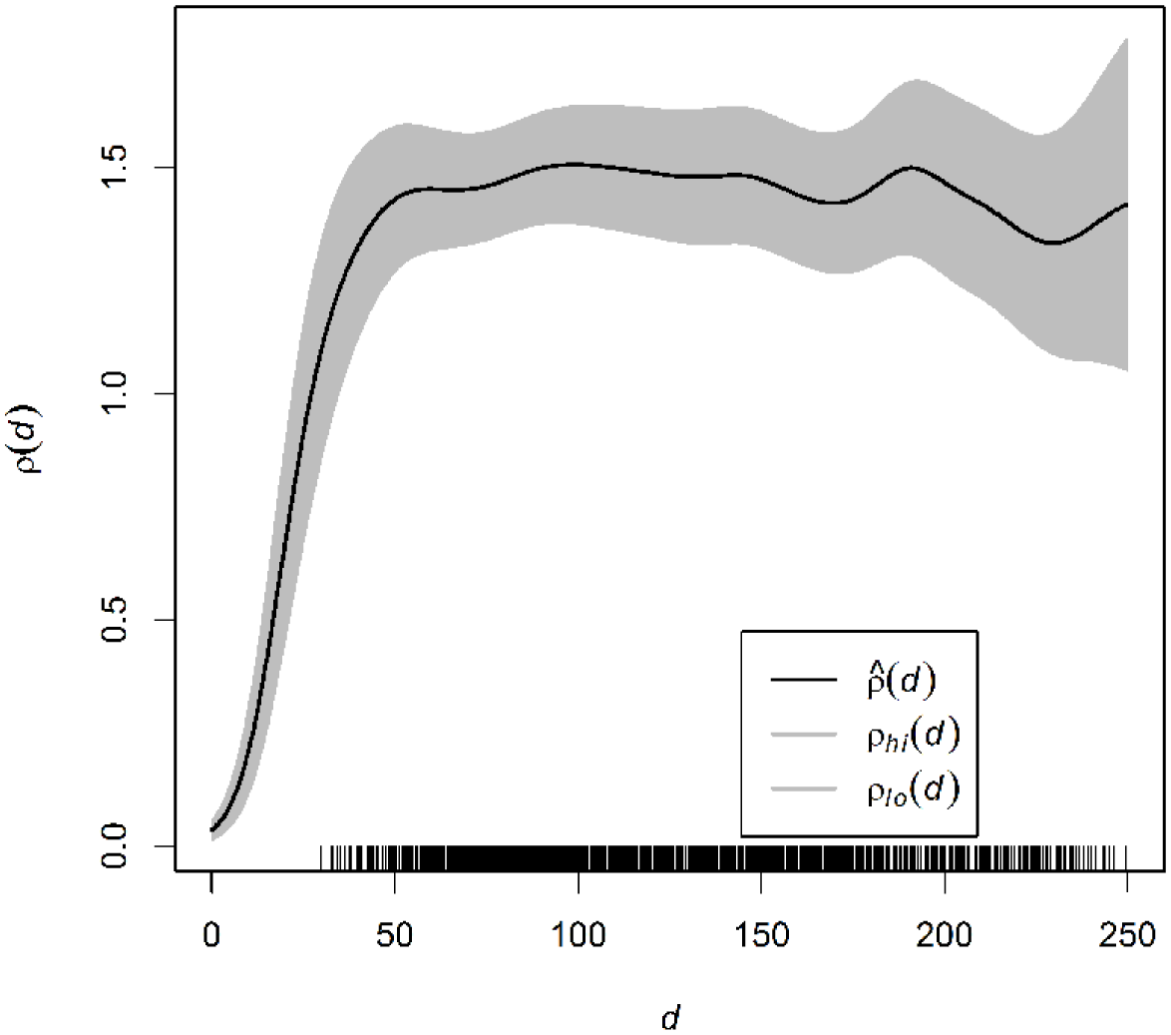
Estimate of the intensity of the first basal arborization of Neuron 1 as a function of the distance (in *μ*m) to the tree root.

The first property to be analyzed is the intensity or average density of points along the network. Spatial inhomogeneity can be conflated with clustering between points. Therefore, it is important to analyze any evidence of spatial variation in point intensity. Indeed, the distribution of dendritic spines along the dendrites of pyramidal cells has been shown not to be uniform in different cortical areas and species (reviewed in [29]). We defined the distance function to the tree root *r* by the shortest path in the dendritic network *d*_*L*_(*u*, *r*) = *d*_*L*_(*u*), *u* ∈ *L*, and we analyzed the relationship λ(*u*) = *ρ*(*d*_*L*_(*u*)), where *ρ* is an unknown function to be estimated. Kernel smoothing methods can be used to estimate the intensity function as discussed in [19, 30]. Fig 4 shows the kernel-smoothing estimate of function *ρ* of the first basal dendrite (see Table 2), confirming that spine intensity depends on the distance to the cell body when it is analyzed along the complete network. The results for all analyzed dendritic networks for both basal and apical dendrites were very similar.

We used the cumulative distribution function (CDF) test to study the hypothesis of independence of intensity on a spatial covariate (distance to the cell body, in our case). The CDF test was first described in [31](in the context of spatial data, using Kolmogorov-Smirnov statistic). For a linear network, the test compares the observed distribution of the values of the covariate in the network events with the null distribution of the covariate at random points on the network. For all analyzed apical and basal dendritic networks, we found strong evidence of the dependence of spine intensity on distance to the cell body (a *p*-value <10^−6^ was obtained in the CDF test in 90% of the cases, the highest *p*-value = 0.00591 being in one of the basal dendrites of Neuron 2).

**Fig 5:**
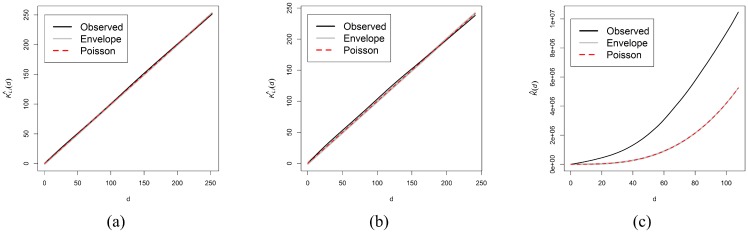
5% critical envelopes of the first basal arborization of Neuron 1. (a) Estimation of 3D geometrically corrected inhomogeneous *K*_*LI*_ function. (b) Estimation of 2D geometrically corrected inhomogeneous *K*_*LI*_ function. (c) Estimation of 3D *K* function ignoring the network (the envelope is just below the red dotted line).

In view of the above results, we fitted an inhomogeneous Poisson model for each dendritic network, in which the spine intensity λ(*u*), *u* ∈ *L* depends on the distance to the cell body. Considering the shape of function *ρ* (Fig 4), we decided to adjust a log-quadratic intensity in *d*, that is, λ(*u*) = exp(*θ*_0_ + *θ*_1_ *d*(*u*) + *θ*_2_ *d*(*u*)^2^), where *θ*_0_, *θ*_1_, *θ*_2_ are the parameters for estimation. Fig 5a shows the estimation of the geometrically corrected 3D inhomogeneous *K*_*LI*_ function (Eq (3)) for the basal example and 5% critical envelopes based on 19 simulations of an inhomogeneous Poisson process with log-quadratic intensity in *d*. The chart shows that the spatial distribution of the spines along the network is consistent with an inhomogeneous Poisson process. Fig 5b is analogous to Fig 5a using the 2D implementation of *K*_*LI*_ provided in the **spatstat** package instead. Although the fit is not bad, it is not as good as in 3D where the estimation of the *K*_*LI*_ function is almost completely superimposed on the Poisson function for all distances *d*. Fig 5c shows the result of applying the 3D *K* function to the spines of the same basal arbor, using only spine spatial coordinates and ignoring the network. This figure incorrectly suggests that spines are strongly clustered. The error stems, however, from the choice of a mistaken null hypothesis because the envelopes are computed from 3D CSR simulations without considering the network.

The results were similar for all analyzed dendritic trees, suggesting that there does not appear to be any evidence of clustering or regularity of dendritic spines after considering spatial inhomogeneity. For three of the analyzed basal networks, however, the estimation of the *K*_*LI*_ function lay slightly below the lower boundary of the envelope at long distances, indicating that there were fewer points within distance *d* of an arbitrary point than within an inhomogeneous Poisson process with log-quadratic intensity in *d*. Conversely, the *K*_*LI*_ function estimations of two of the apical networks remained outside the envelope at long distances but above the upper boundary of the envelope, suggesting that points tended to be closer than within an inhomogeneous Poisson process at long distances. This could mean that spine distribution differs slightly in basal and apical arborizations as the distance to the cell body increases. Then we used the studentized permutation test to analyze if there were differences between different pyramidal neurons and between basal and apical dendrites.

**Fig 6:**
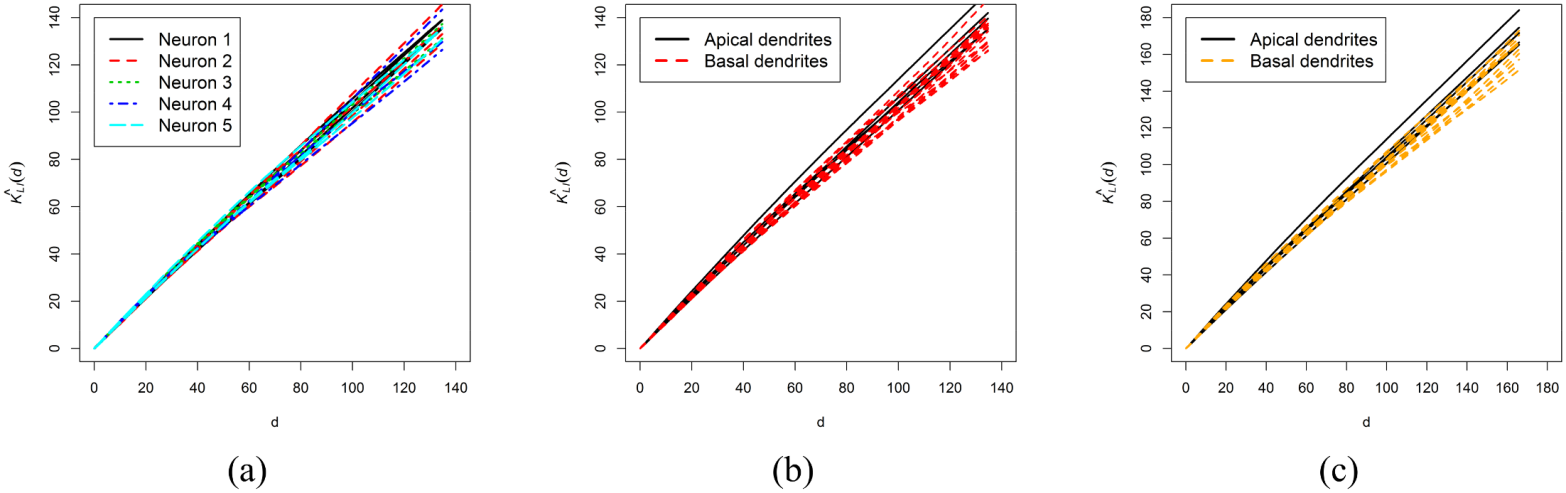
Estimated 3D *K*_*LI*_ functions used in the studentized permutation test. (a) Estimated 3D *K*_*LI*_ functions of all basal networks grouped by neuron in the distance range [0, 134.70] (*g* = 5 groups, *p*-value = 0.808). (b) Estimated 3D *K*_*LI*_ functions of all apical networks forming a group and all basal networks forming another group in the distance range [0, 134.70] (*g* = 2, *p*-value = 0.109) (c) Estimated 3D *K*_*LI*_ functions of all apical networks forming a group and the basal dendrites of Neurons 1, 3, 4 and 5 forming another group in the distance range [0, 165.96] (*g* = 2, *p*-value = 0.045).

First, we compared groups of basal arborizations of different neurons, that is, we applied the test with *g* = 5 groups (neurons) using their previously estimated 3D *K*_*LI*_ functions in the range of distances [0, 134.70]. We used a maximum distance that was 2% lower than the minimum *circumradius* *R* of all the networks used in the test. We obtained a *p*-value of 0.808. Thus, we concluded that there were no significant differences in spine distributions along the basal trees among the five neurons at the analyzed distances (Fig 6a). Then, we applied the test again, forming a group with all basal arborizations of the five pyramidal neurons and another group with all apical arborizations. The resulting *p*-value was 0.109. Therefore, we concluded that there were no statistically significant differences between spine distributions of these two groups up to a distance of 134.70 *μ*m (Fig 6b).

We wanted to analyze if there were differences in spine distribution between basal and apical dendrites taking into account distances farthest from the cell body. In the studentized permutation test, each group should contain at least three patterns to achieve reasonably precise estimates for the within-group variance of the estimates. To do this, we decided to remove Neuron 2 from the analysis because two of its basal trees had a small *circumradius* (137.46 *μ*m and 139.79 *μ*m, respectively), and we repeated the analysis with all the other neurons up to a distance of 165.96 *μ*m (distance that was 2% shorter than the minimum *circumradius* *R* of the remaining basal networks). First, we compared groups of basal arborizations. We obtained a *p*-value of 0.565 for *g* = 4 groups (Neurons 1, 3, 4 and 5). Therefore, we concluded that there were no significant differences in spine distribution along basal trees in the range of distances [0, 165.96] either. We applied the test again, forming a group with the 13 basal dendrites analyzed in the previous step, and another group with all apical dendrites (all with a *circumradius* longer than 165.96 *μ*m). We obtained a *p*-value of 0.045, and, with the usual 5% significance level, we concluded that, contrary to previous cases, there were significant differences in spine distribution along apical and basal dendritic networks considering distances up to 165.96 *μ*m. Fig 6c suggests that apical dendritic spines are more clustered than basal dendritic spines as the distance from the cell body increases.

## Discussion

We analyzed the spatial distribution of spines along both basal and apical dendritic networks of human pyramidal neurons. To do this, we used network spatial analysis, implementing methods to analyze 3D linear networks for the first time. We studied whether there were differences in the spatial distribution of spines between different pyramidal neurons and between basal and apical dendrites, using replicated point patterns in conjunction with network spatial analysis. To do this, we took advantage of the geometrically corrected *K* function in order to compare the corrected *K* functions obtained from different point patterns in different networks [22].

A non-constant intensity of points can be easily confused with clustering between points. Therefore, we set out to thoroughly analyze spine intensity in dendritic networks. We found that there was spatial variation in spine intensity which depended on the distance to the cell body. Therefore, we fitted an inhomogeneous Poisson model. The model used appeared to adequately explain the spatial distribution of spines along dendritic networks in most cases. Additionally, we found that there were no significant differences in spine distribution between basal trees of the same and different neurons. This suggests that dendritic spine distribution in the basal dendritic arbors conforms to common rules. Neither did we find statistically significant differences between basal and apical trees up to distances of 134.70 *μ*m away from the cell body. Excluding the smaller basal networks and analyzing distances farthest from the cell body (up to 165.96 *μ*m), however, we did find significant differences in the distribution of spines along basal and apical networks. The spines of apical dendrites are more clustered than basal spines. Therefore, not only do apical and basal dendritic arbors have distinct morphologies, but the rules of spine distribution are also different. These observations further emphasize that synaptic input information is processed differently within these two dendritic domains. Note, however, that, as stated in [5], the analysis performed may be very sensitive to the fitted intensity, especially in tree-like networks. Because of this, it might be interesting to examine other models that further characterize the spatial distribution of spines along the basal and apical networks, especially at distances further from the cell body.

Recent research analyzing the distribution of spines along dendritic networks yielded different results. In [4] it is concluded that spine intensity is completely spatially random. In [5], where only one example pattern of the cells analyzed in [4] is studied, it is found that different branches may have different patterns of spine distribution. The dendrites investigated in these studies belong to cell culture *in vitro* rat dissociated hippocampal neurons, while we analyzed adult human neocortical pyramidal cells obtained at autopsy. Thus, differences in spine distribution are not comparable because of possible differences between human and rat pyramidal cell structures, as well as between the experimental approaches used to obtain the tissue, neuron labeling and methods of analysis.

This is the first paper to take into account the third dimension of spatial analysis on linear networks. This approach has been applied to the example of spines along dendritic networks but could be useful for the spatial analysis of other real-world 3D networks. The shortest path distances in the network are much harder to compute than Euclidean distances in traditional spatial statistics. Besides, the inclusion of the third dimension considerably increases the computational load especially with increased network complexity. As future work, we would like to improve the efficiency of the implementation developed for 3D networks. Also, it would be interesting to consider the network (dendrite) volume and the possibility of events (spines) occurring on the surface of the network with volume. In this case, 3D analysis could be even more useful, although the methodology would need to be expanded. The inclusion of marks in the analysis, such as some spine characteristics like length, volume or type [16, 32], may also be beneficial for elucidating important aspects of the spatial distribution of spines. Finally, alterations of spine distribution are common in the diseased brain (for a review see [33]). Thus, the analysis performed in this study may shed light on the possible alterations of neuronal circuits in brain diseases.
